# Acknowledgement to reviewers

**DOI:** 10.1530/ETJ-14-A1

**Published:** 2025-02-19

The editorial board wishes to thank the following individuals who have helped to review manuscripts for *European Thyroid Journal* during 2024. Their assistance is gratefully acknowledged.

S Adam

M Alevizaki

C Alvarez

P A G Alves

S L Andersen

M Andersson

T E Angell

K Ashida

M Aung

J H Baek

S Balasubramanian

B Ballieux

Z W Baloch

S Bárez-López

L Bartalena

I Başara Akın

T Bednarczuk

C Bellevicine

A Ben Hamou

I M Bensenor

A Bianco

A Bikas

B Biondi

L Blankers

K Boelaert

A Boelen

J Boer

S Bonnema

F Borson-Chazot

A Botezatu

A Bracke

G Brigante

C Buffet

D A Bulzico

H B Burch

M Burlacu

K D Burman

B Cakir

M Cakir

J Calissendorff

I Campi

A R Cappola

P Caron

M G Castagna

F S Celi

M Centanni

K Chatterjee

T Cheeetham

T Cheetham

N Chevalier

K K Chong

R J Clifton-Bligh

M C Coelho

C Colombo

B Corvilain

C A Cuevas Rodríguez

C Daumerie

C E Daumerie

L Davies

C Dayan

J de Filette

S De Leo

M Debono

B Decallonne

M Decaussin-Petrucci

J Deladoey

M Dentice

A Derakhshan

Y Dotsikas

L Duntas

C Durante

G Effraimidis

R Elisei

C Ellervik

M F Erdogan

A Ferlito

C Ferraz

F Flamant

E Fliers

A Frasoldati

S Frey

L Fugazzola

T Fukuhara

D Gallo

J Galofré

V A Galton

F Gatto

B Gereben

A Gianoukakis

B Gips

A R Glover

B Goichot

G Grani

M L Guia Lopes

M Gurnell

S Hanna

D Hartl

J V Hennessey

S Hescot

T Hiromasa

S Hoenes

P Hunt

F Illouz

A Imperiale

Y Ito

C Johns

C C Juhlin

C K Jung

G J Kahaly

T Kawamoto

B Khoo

W G Kim

A Kjaergaard

J Knauf

A Kober

M Kocova

J Koehrle

E Kornelius

A Kotwal

A Kus

M Kwon

L Lamartina

I Landa

G Lanzolla

F Latrofa

L J Leandro-Garcia

S Leboulleux

J S Lee

J Y Lee

S Lee

A M Leung

Q Lian

H Lin

T P Links

Z Liu

M Losa

Y Luk

D Luo

M A Lupo

A L Maia

N Makita

Z Maleki

J Mammen

A Maniakas

M Marino

C B Marshall

H Masuoka

A Matrone

S Mayerl

S Mayilvaganan

M Medici

C Mian

K Minamitani

L Möller

S Monteiro-Martins

R Moreno-Reyes

H Mori

C Muir

I Muller

J Nagarajah

M Nakano

R Netea-Maier

M Niedziela

A Nikitski

E Nishihara

D V Nostrand

H F Nyström

K Ohba

A Olivieri

J Osinga

N Otsuki

A Ozpinar

F Pani

T Pappa

J J Park

S Pearce

B Pekova

G Pellegriti

P Perros

L Persani

L Petrick

R Pichler

A Piekiełko-Witkowska

F Pitoia

K G Poppe

J Prasuhn

M Puig-Domingo

E Puxeddu

S Razvi

J Riou

E Robenshtok

P W Rosario

I L Ross

E Rossi

R M Ruggeri

G Russ

P Saeed

N Sakata

D Salvatore

M Salvi

A Sanabria

N Satoshi

L Schomburg

C Selmer

D Sharlin

Y Shibata

N Singh Ospina

R Sinha

J Sosa

M Sponziello

A Stoupa

I Stramazzo

K Sugino

I Sugitani

K Swan

Y Takahashi

Y Takeuchi

M Tanda

F Tessler

A Teumer

P Thuillier

M Tian

C Tomoda

E Torlone

M Trevisan

P Trimboli

F Triponez

M Tuttle

M Urken

B Vaidya

F Vaisman

A van der Spek

A van Herwaarden

P van Houten

A S van Trotsenburg

E van Velsen

W E Visser

J P Walsh

Y Wang

J D Wasserman

A J Wassner

C Wenzek

E K Wirth

M Xing

W Xu

A Yoshihara

H K L Yuen

C Zafon

W Zandee

M Žarković

M C Zatelli

W Zhang

X Zhu

